# Reverse Electrodialysis-Assisted Solar Water Splitting

**DOI:** 10.1038/s41598-017-12476-3

**Published:** 2017-09-25

**Authors:** Jihye Lee, Jeongse Yun, Seung-Ryong Kwon, Woo Je Chang, Ki Tae Nam, Taek Dong Chung

**Affiliations:** 1 Department of Chemistry, Seoul National University, Seoul, 08826 Korea; 2 Department of Materials Science and Engineering, Seoul National University, Seoul, 08826 Korea; 3 Department of Materials Science and Engineering, Northwestern University, Evanston, Illinois 60208 USA; 4 Advanced Institutes of Convergence Technology, Suwon-si, Gyeonggi-do, 16229 Korea

## Abstract

Photoelectrochemical (PEC) water splitting provides an attractive route for large-scale solar energy storage, but issues surrounding the efficiency and the stability of photoelectrode materials impose serious restrictions on its advancement. In order to relax one of the photoelectrode criteria, the band gap, a promising strategy involves complementing the conventional PEC setup with additional power sources. Here we introduce a new concept: solar water splitting combined with reverse electrodialysis (RED). RED is a membrane-based power generation technology that produces an electrochemical potential difference from a salinity gradient. In this study, the RED stack serves not only as a separator, but also as an additional tunable power source to compensate for the limited voltage produced by the photoelectrode. A hybrid system, composed of a single-junction p-Si and a RED stack, successfully enables solar water splitting without the need for an external bias. This system provides flexibility in photoelectrode material selection.

## Introduction

Global energy consumption and environmental issues have led to an increasing demand for environmentally friendly and sustainable energy systems. The attention given to solar energy is, in large part, due to the tremendous amount of solar power available on Earth^1^, despite some intrinsic issues such as its diffuse nature^1^, and its dependence on environmental conditions such as weather and climate^2^. Integrating different resources to form a suitable hybrid system can provide a remarkable way for improving efficiency and reliability^2,3^. This is mostly because hybrids can provide the complementary benefits of the respective source materials^4^.

Photoelectrochemical (PEC) water splitting provides a prominent route for large-scale solar energy storage by generating a useful chemical fuel, *e.g*., gaseous hydrogen^5^. In a PEC cell, the most important element is the photoelectrode, where the incident light is converted into electrical energy, and where the water-splitting reaction takes place to produce hydrogen fuel^5^. There are several essential requirements for photoelectrode materials. Firstly, the photoelectrode must have a suitable band gap. The photoelectrode must generate a photovoltage sufficient for the electrochemical reduction and oxidation of water, while utilizing a large portion of the solar spectrum. Secondly, the band-edge potentials at the surface must be more negative and more positive than the reduction and oxidation potentials of water, respectively. Thirdly, charge transfer at the semiconductor/electrolyte interface must be so rapid that charge carriers are not consumed by recombination. Finally, the photoelectrode must be stable, while operating over a long period of time in aqueous electrolytes^6^. Over several decades, a great deal of effort has been directed toward the identification of suitable photoelectrode materials including tandem configuration^7–10^, band edge engineering^11,12^, synthesis of new materials and/or modification of existing materials^13–15^, surface passivation^10,16–19^, catalysts^20–25^. But a material that satisfies all the above requirements has not yet been reported^6^.

Silicon is one of the most widely used materials for both photovoltaic and PEC cells because of its earth abundance and low cost. In addition, the band gap (1.1 eV) of Si is suitable for absorption over a broad range of the solar spectrum, which contributes to high theoretical photocurrents and solar fuel conversion efficiencies^26^. However, the intrinsically low photovoltage associated with the narrow band gap of Si is a major challenge for unassisted solar water splitting. In the case of a single-junction configuration, the maximum obtainable photovoltage for Si is 0.8 V, while the thermodynamic voltage required for water splitting is 1.23 V^26^.

Here, we introduce a unique solar water-splitting system composed of a PEC cell equipped with an additional voltage source, namely a reverse electrodialysis (RED) stack. The RED stack is an assembly of alternating cation- and anion-exchange membranes (CEMs and AEMs) that allow charge-selective ion transport. When solutions with different ion concentrations flow on opposite sides of an ion-exchange membrane, an electrochemical potential difference develops across the membrane^27^. According to Nernst equation, an ion-exchange membrane can create a potential difference of about 90 mV at a salinity ratio of 50 (for more details see Supplementary Fig. S1)^28^. Several pairs of high-concentration (HC) and low-concentration (LC) solutions are alternatively stacked to generate a unidirectional electric field. An overall potential difference, from one end of the RED stack to the other, is created in proportion to the number of membranes in the series (for more details see Supplementary Fig. S1).

**Figure 1:**
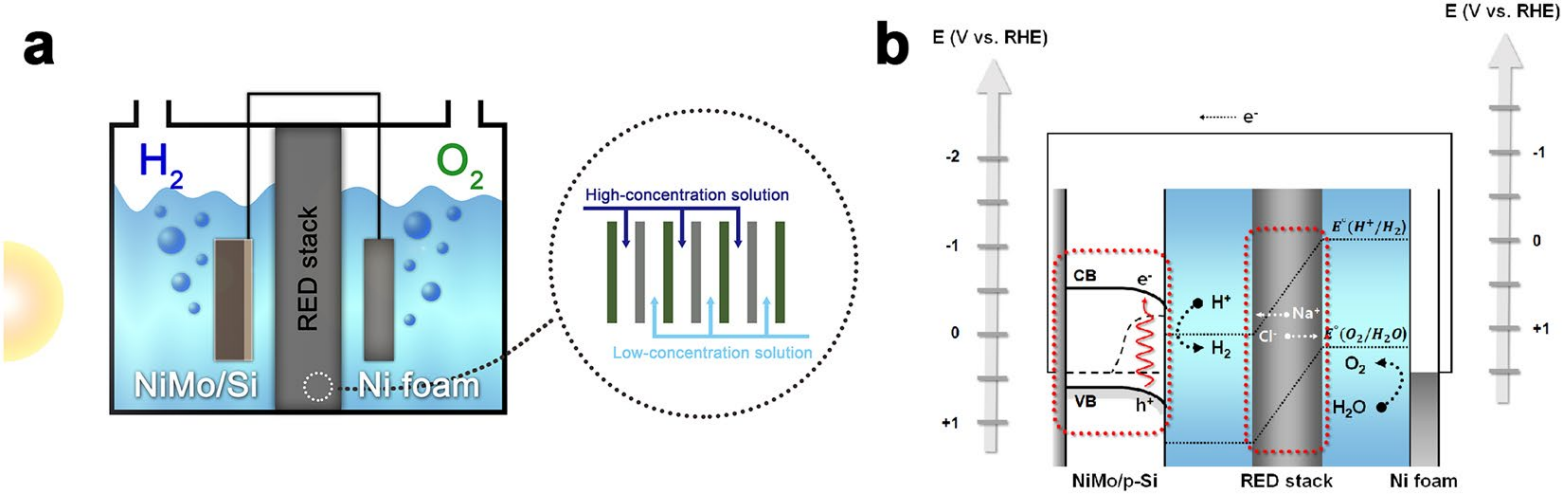
RED-assisted solar water-splitting system composed of a p-Si photocathode, a Ni foam anode, and a RED stack. (**a**) Schematic diagram of the integrated PEC-RED system. The RED stack consists of several pairs of cation- (green) and anion-exchange membranes (gray). (**b**) Energy diagram of the system. Red-dotted boxes indicate two power sources. CB and VB mean the conduction and valence bands of p-Si. Black dotted line in Fig. 1b represents the quasi-Fermi levels of electrons and holes, which is generated under illumination. ${E}^{^\circ }({{\rm{H}}}^{+}/{{\rm{H}}}_{2})$ and ${E}^{^\circ }({{\rm{O}}}_{2}/{{\rm{H}}}_{2}{\rm{O}})$ are water reduction and oxidation potentials, respectively.

In general, a membrane that separates the catholyte from the anolyte in the water-splitting cell, *e.g*., Nafion, is essential for assuring electrical conduction under conditions of minimal contamination by chemical products in each solution^29^. However, the membrane can increase the solution resistance, or cause significant pH gradients near the electrode surface, resulting in a severe reduction in system efficiency^29^. In the PEC-RED hybrid system, a RED stack between the electrodes can not only serve as a separator for preventing product crossover, but also as an additional voltage source to subsidize photovoltaic power that may be insufficient for solar water splitting. By integrating a single-junction Si photoelectrode with a RED stack that is a substitute for a conventional proton-selective membrane, *e.g*., Nafion, we successfully enables solar water splitting without any external bias, as shown in Fig. 1a. This approach revives a number of narrow band gap semiconductors as candidates for photoelectrode materials in solar water-splitting systems, while maintaining the high photocurrent of the narrow band gap material.

## Results and Discussion

### Preparation and characterization of electrodes

One of the challenges for efficient solar water splitting is the sluggish kinetics for reactions that take place at the semiconductor surface. In order to facilitate interfacial charge transfer, electrocatalysts such as platinum, ruthenium oxide, or iridium oxide should be coupled with semiconductor electrodes. NiMo is an Earth-abundant metal electrocatalyst that shows remarkable activity and stability for hydrogen evolution reactions (HER)^30^. In this work, NiMo was electrochemically deposited on a p-Si photocathode under intense irradiation. Supplementary Fig. S2 shows the morphology of a NiMo electrocatalyst film deposited on a p-Si surface. The NiMo film, as prepared, was discontinuous and comprised of porous multilayers that appear to be clusters of particles. This morphology seems to result from the co-evolution of H_2_ bubbles during the deposition of NiMo^31^. Earth-abundant electrocatalysts, including NiMo, require large mass loadings to achieve catalytic activities comparable to that of noble metal catalysts, due to their intrinsic low specific activities^20^. However, significant loss of incident light is unavoidable because Earth-abundant electrocatalysts are generally optically opaque^24^. It is therefore necessary to find a balance between catalytic activity and optical transparency. We determined the optimal time for NiMo electrodeposition, as shown in Supplementary Fig. S3. The best film thickness was determined to be about 3.3 μm (for more details see Supplementary Fig. S2). X-ray photoelectron spectroscopy (XPS) and X-ray fluorescence spectroscopy (XRF) confirmed the deposition of NiMo. The Ni:Mo ratio was determined to be approximately 86:14, which is similar to that for previously reported compositions (for more details see Supplementary Fig. S2)^31^.

**Figure 2:**
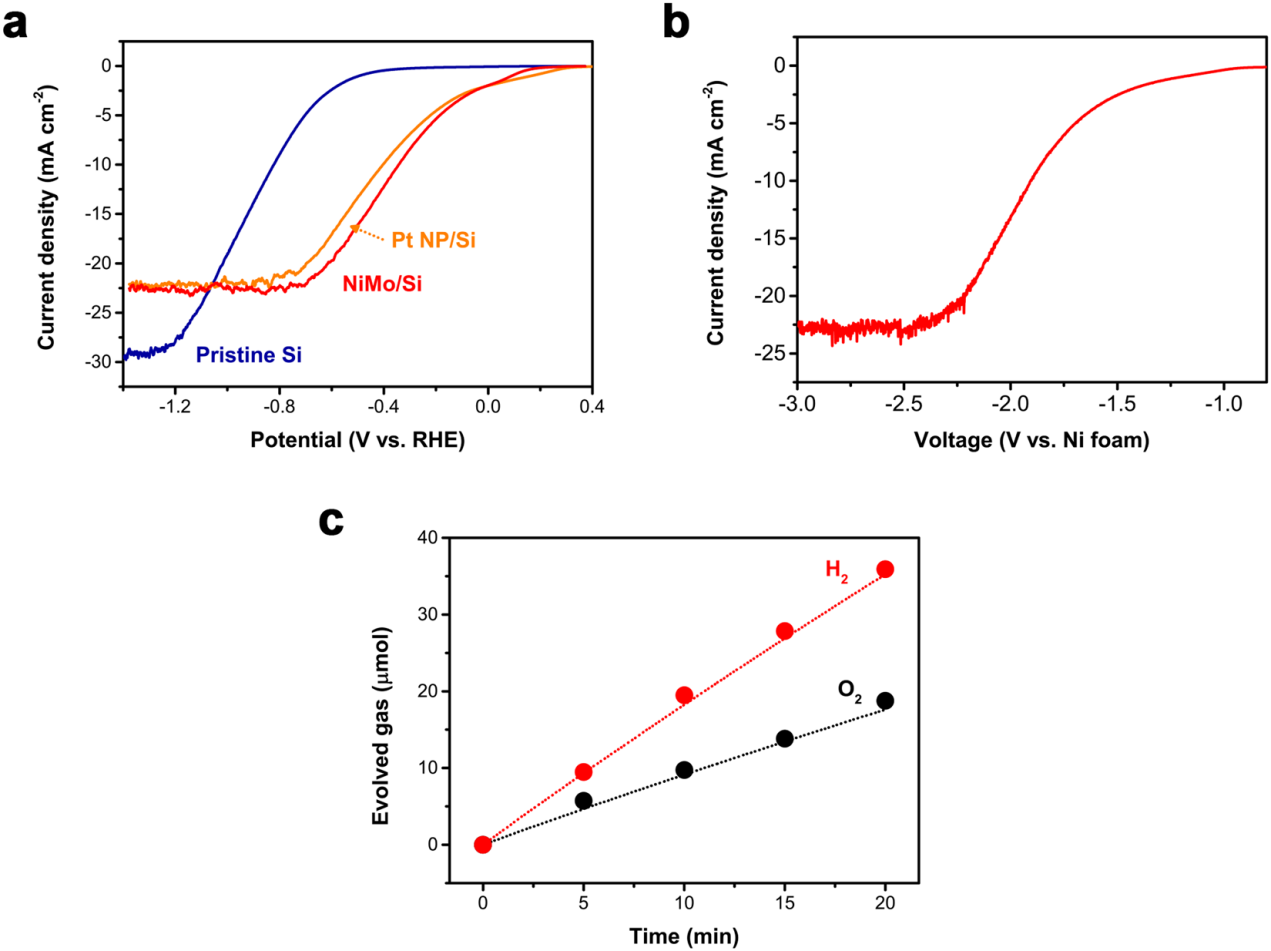
The electrochemical performance of a standalone PEC cell. (**a**) Hydrogen evolution reaction at NiMo/Si (red), Pt NP/Si (orange), and pristine Si (blue). (**b**) Overall water-splitting performance. NiMo/Si and Ni foam electrodes are connected in a two-electrode configuration. (**c**) Evolution of H_2_ and O_2_ gas at NiMo/Si and Ni foam electrodes. Gas measurement was carried out by applying a voltage of 2.65 V across the electrodes. Faradaic efficiency approached to 100%. The theoretical H_2_ (red line) and O_2_ (black line) amounts was calculated based on the charge passed during electrolysis. All electrochemical experiments were conducted in 0.1 M phosphate buffer (pH 7.0) under simulated AM 1.5 G irradiation (100 mW cm^−2^).

The photoelectrochemical catalytic activity of the NiMo/Si photocathode was characterized in 0.1 M phosphate buffer (pH 7.0) under simulated AM 1.5 G irradiation (100 mW cm^−2^) (Fig. 2a). The HER performance of NiMo/Si proved to be comparable to that of Pt nanoparticles deposited Si (Pt NP/Si, orange), and much better than that of pristine Si (blue). Although the NiMo catalyst has a significantly low specific activity for HER, electrodeposited NiMo could exhibit a similar geometric activity to that of Pt, due to a large electrochemically active surface area^20^. NiMo electrocatalysts with large surface roughness values have been shown to exhibit higher HER activities than Pt in alkaline electrolytes^20^. The NiMo/Si photocathode was used to investigate water-splitting performance.

For the oxygen-evolving anode, we chose Ni foam. Nickel (Ni) is widely used for commercial alkaline electrolyzers owing to its Earth abundance and excellent corrosion-resistance^32^. Oxygen evolution at Ni foam, in 0.1 M phosphate buffer (pH 7.0), requires a high overpotential indicating slow electrokinetics at the electrode surface, as shown in Supplementary Fig. S4. Figure 2b displays the overall water-splitting performance of the (NiMo/Si and Ni foam) two-electrode system in 0.1 M phosphate buffer (pH 7.0). In spite of good kinetics at the NiMo cathode, overall water splitting requires at least 1.2 V of extra potential difference to achieve a current density of 1 mA cm^−2^. To reach the photo-limited current density at the NiMo/Si electrode (23 mA cm^−2^), the two-electrode system requires more than 2.5 V of external supply in addition to the photovoltaic driving force.

In most cases, water splitting is conducted in either a highly acidic or a highly alkaline electrolyte. This minimizes voltage losses associated with concentration overpotentials that are related to mass transport limitations, as well as the kinetic overpotentials required of the electrocatalyst during the water-splitting reaction. However, most semiconductors and Earth-abundant electrocatalysts are unstable under such harsh pH conditions. For example, Si is relatively stable in acidic electrolytes, but is very vulnerable to corrosion under alkaline conditions^26^. Similarly, NiMo shows excellent catalytic activity and stability in alkaline electrolytes, but exhibits poor stability in strong acids^26,33^. Therefore, it is crucial to operate the water-splitting system at near-neutral pH, in order to utilize a greater variety of electrode materials^29,34^. However, operating at neutral pH involves a substantial voltage penalty because of large overpotentials and severe pH gradients^29,34–36^. This is a further reason why such a large overpotential is required for water splitting, as shown in Fig. 2b.

The evolved H_2_ and O_2_ that were obtained when a 2.65 V potential difference was applied across the NiMo/Si and Ni foam were quantified by gas chromatography (GC). GC analysis confirmed that hydrogen and oxygen gases are evolved in a 2:1 ratio with a faradaic efficiency close to 100% (Fig. 2c).

### Characterization of RED performance

**Figure 3:**
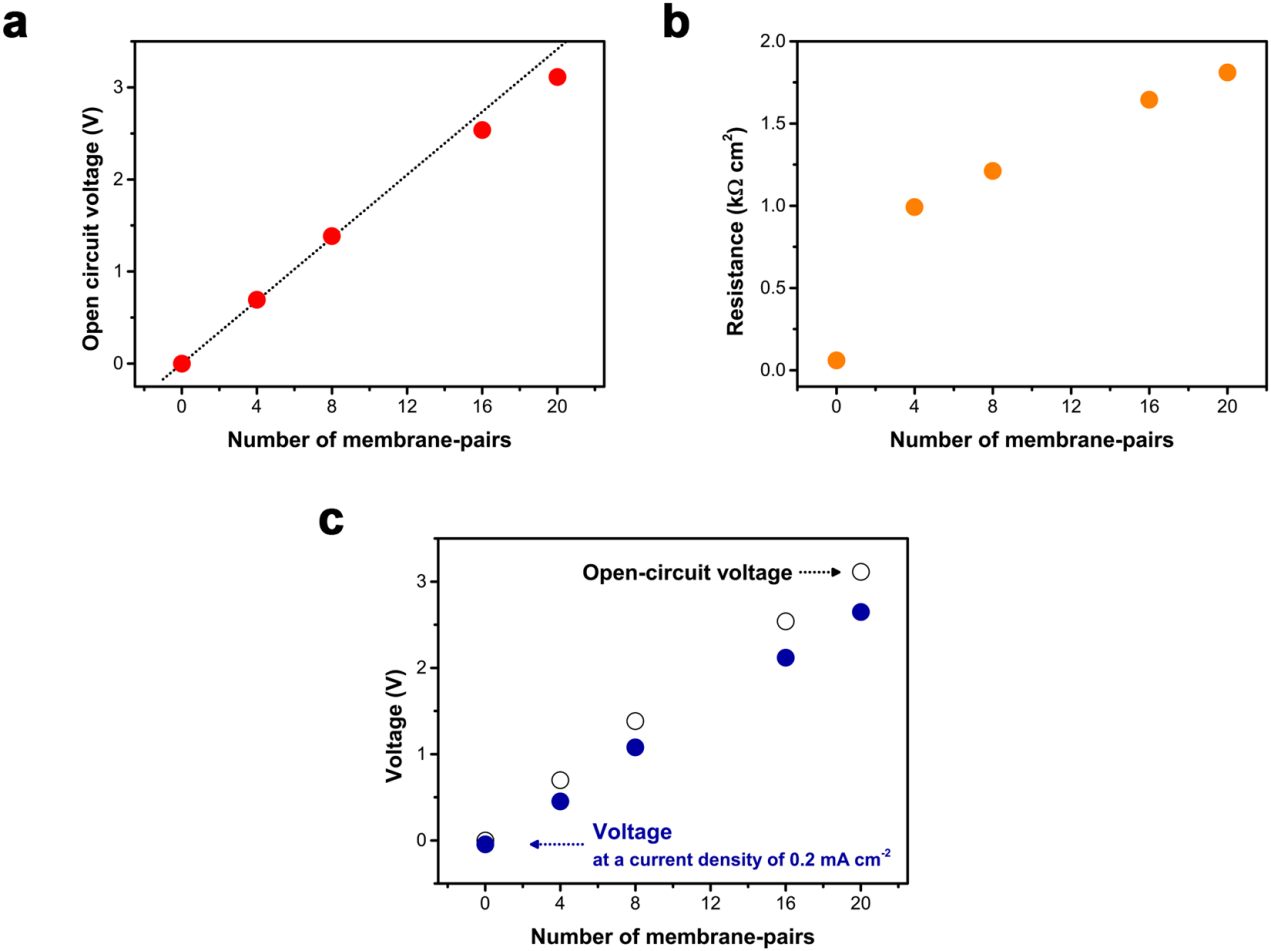
The electrochemical performance of the RED stack. (**a**) Open-circuit voltage (OCV) produced from the RED stack under open-circuit condition. Experimentally measured OCV values (red) show excellent agreement with theoretical OCV values (black line), calculated from the Nernst equation. (**b**) Resistance at the RED stack. (**c**) Available voltage for the faradaic reaction at electrodes (solid). Difference between OCV (open) and available voltage (solid) indicates a loss in potential associated with the RED stack.

In this study, we utilized the RED stack to obtain an additional bias. The voltage produced from RED stacks under open-circuit conditions is presented in Fig. 3a. The open-circuit voltage (OCV), *i.e*., the maximum voltage that can be obtained from the RED stack, increases linearly, by about 0.15 V per membrane-pair (CEM and AEM). The experimentally measured OCV values are in good agreement with those predicted theoretically by the Nernst equation (for more details see Supplementary Fig. S1)^28^.

For efficient water splitting, the potential losses arising from electrode overpotentials, solution resistance, and membrane resistance, should be minimized^36^. Accordingly, most of the potential drop should occur at the electrode-electrolyte interface, where the faradaic reactions take place in conventional PEC system. In our system, the RED stack participates in the circuit, not only as a voltage source, but possibly also as a resistor. The resistance of the RED stack consists of both ohmic and non-ohmic components. The ohmic component is produced by membrane resistance and the limited conductivity of the HC and LC solutions. The non-ohmic resistance results from changes in the ion-concentration distribution near a membrane surface when an electrical current flows^37^. To determine the resistance associated with the RED stack, an electrochemical impedance analysis was carried out at a current density of 0.2 mA cm^−2^ (normalized to the membrane area) using two Ag/AgCl electrodes (Fig. 3b). Resistance increases steeply when the RED stack with 4 membrane-pairs is inserted between the electrodes. This observation is associated with the structure of the RED stack (for more details see Supplementary Fig. S1). At both ends of the RED stack, there are wide compartments into which HC and LC solutions are injected. Among them, the wide, LC solution-filled compartment contributes most strongly to the steep increase in total resistance of the RED stack. This is because the electrical resistance of the stack is dominated by the dilute LC solution with low conductivity^38^. To clarify the effect of LC on the electrical resistance of the RED stack, the stack resistance as a function of flow rate, was also measured (for more details see Supplementary Fig. S5). At higher flow rates, a high salinity gradient is maintained throughout the stack, providing higher OCV and resistance. Although the thickness of the diffusion boundary layer, and the resistance associated with it, decreases at higher flow rate, an increase in the membrane resistance itself and the low conductivity of the LC solution are the main factors responsible for the high resistance^38–40^. As the flow rate is reduced, the HC and LC solutions reside for longer inside the stack. As a consequence, the average salinity difference gradually decreases, resulting in lower OCV and resistance.

Figure 3c shows the OCV (open circle), and the voltage across the RED stack under the galvanostatic condition of 0.2 mA cm^−2^, corresponding to the photo-limited current density at the NiMo/Si electrode (solid circle). The difference between these voltages corresponds to the voltage drop that occurs across the RED stack with maximal NiMo/Si current flowing. Only a small potential loss is associated with the RED stack itself, indicating that most of the voltage obtained from the RED stack is used to assist faradaic reactions at the electrode. Therefore, RED can serve as an effective additional power source.

### Integrated water-splitting cell

Through the combination of a PEC cell and a RED stack, an overall water-splitting cell was constructed in a two-electrode configuration. A schematic diagram of the PEC-RED system is illustrated in Fig. 1a. A photograph of the experimental set-up is presented in Supplementary Fig. S6. As discussed above, we used NiMo/Si and Ni foam as the hydrogen- and oxygen-evolving electrodes, respectively. The RED stack, situated between the two electrodes, acts as an additional power source, as well as a salt bridge that separates the catholyte from the anolyte. A simplified energy diagram of the cell is depicted in Fig. 1b (for more details see Supplementary Note). The electric circuit has two voltage sources, *i.e*., the Si photoelectrode and the RED stack, that are connected in series. For p-Si, the potential of the valence band is not sufficiently positive for water oxidation, resulting in negligible overall water splitting. By integrating the RED stack with the p-Si photoelectrode in series, the voltage from the RED stack is added to the photovoltaic potential difference from the p-Si, providing sufficient power for water splitting.

**Figure 4:**
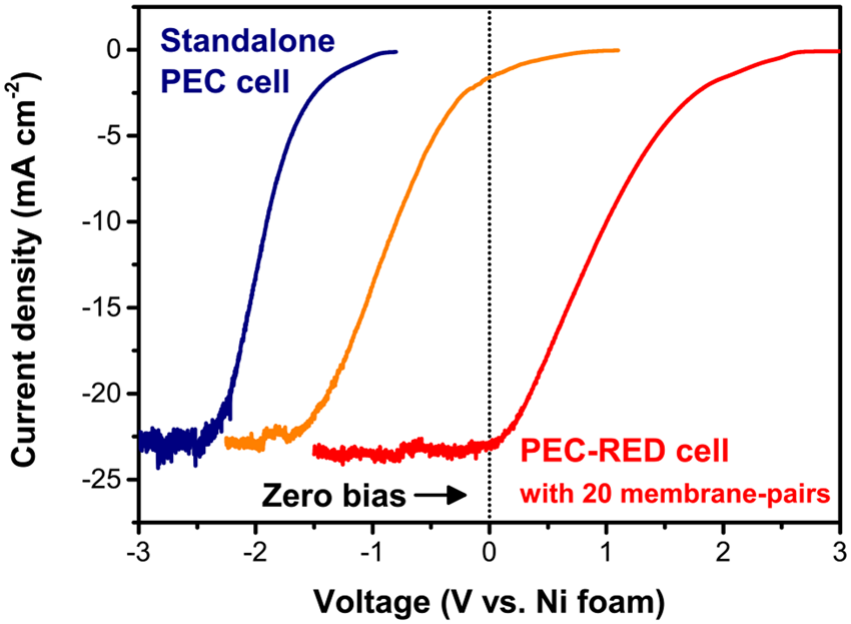
Overall water-splitting performance of the integrated PEC-RED system. A standalone PEC cell (blue). Integrated PEC-RED cells with 8 (orange) and 20 (red) membrane-pairs. With the RED stack, the current density-voltage curves shift in the positive direction. At the same time, the water-splitting reaction is initiated and the current density increases up to 23 mA cm^−2^ without any external bias.

As mentioned above, overall water splitting is negligible in the PEC system alone. An external bias of at least 1.2 V is required to achieve a current density of 1 mA cm^−2^, and more than 2.5 V is needed to reach the photo-limited current density of NiMo/Si. RED stacks with 8 and 20 membrane-pairs correspond to 1.4 V and 3.1 V under open-circuit conditions, respectively. With these ionic voltage suppliers, the current density-voltage curves shift in the positive direction. At the same time, the water-splitting reaction is initiated and the current density increases to 1.6 mA cm^−2^, and finally up to 23 mA cm^−2^, which corresponds the maximum current density of NiMo/Si (Fig. 4). Currently, there are limited remedies for the photoelectrochemical reaction requiring high photovoltage in a conventional PEC cell. In particular, wider band gap semiconductor materials^41^, and/or tandem photoelectrodes^7,9^ bring about significant reductions in photocurrent. The RED stack serves as both an ionic voltage source and a salt bridge; consequently, it allows us to tune the potential difference without altering the electrode materials. By introducing the proposed power-generating salt bridge, photocurrent density needs not be sacrificed for additional overpotential.

**Figure 5:**
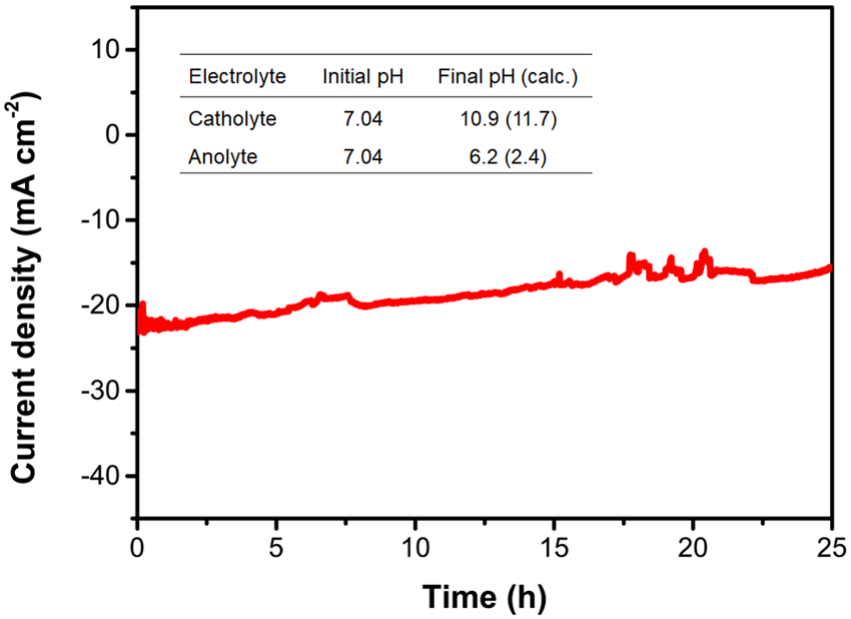
Stability of the integrated PEC-RED system. Overall water-splitting current density for the integrated PEC-RED cell composed of a 24 membrane-paired RED stack, under simulated illumination without any external bias. The inset shows the pH change after 25 h of electrolysis. Calculated pH values, based on the total charge flowing during electrolysis, are provided in parentheses.

Stability is one of the key issues for sustainable energy system implementation. A PEC-RED cell with 24 membrane-pairs displayed a constant current density of about 20 mA cm^−2^ over 25 h, assuring good stability of both the Ni-Mo/Si photocathode and the Ni foam anode, as well as the RED stack (Fig. 5). After water splitting for 25 h, the pH of the catholyte and the anolyte changed from 7.04 to 10.9 and to 6.2, respectively. The pH change induced by water electrolysis can cause problems in terms of the material stability and the operating voltage. After the long-term operation test, the pH of the catholyte increased considerably from 7.0 to 10.9, which could have a detrimental effect on the stability of NiMo/Si. However, it seems that 3 μm thick NiMo serves as a passivation layer for Si as well as a catalyst for water reduction reaction. Moreover, a higher overpotential is needed for water electrolysis as the pH of catholyte increases (+59 mV/pH unit). In the case of the PEC-RED cell with 24 membrane-pairs, the system works at the photo-limited region to be stabilized with respect to small voltage variations. The fluctuations in the Fig. 5 were caused by the formation and dislodge of bubbles on the electrode surface, which were generated by the water splitting reaction. A full-scale RED pilot plant was successfully operated for over five months in a real outdoor environment without any significant performance losses^42^. This indicates that the PEC-RED configuration greatly assists in stabilizing the solar water-splitting system, even when the catalytic activity of the photoelectrode is insufficient.

To calculate the net power produced by the PEC-RED cell, the energy that is consumed to pump the HC and LC solutions into the RED stack needs to be taken into account by subtracting the hydrodynamic loss from the H_2_ chemical power produced^43^. Supplementary Table S1 lists the hydrodynamic losses for the RED stack. At a flow rate is 9 mL min^−1^, the power consumed for pumping is 19% of the total power produced. Given that there is only a small difference in current density at 9 mL min^−1^ under zero bias conditions to that produced at 3 mL min^−1^ (for more details see Supplementary Fig. S7), the power consumed for pumping decreases to 4%, at the lower flow rate, compared to the power produced, which corresponds to a system efficiency of about 1.8% (for more details see Supplementary Table S2).

Based on calculations, after solar water splitting for 25 h, the pH of the anode compartment was expected to decrease to 2.9, but experimentally it only changes from 7.0 to 6.2 (Fig. 5, inset). This observation is related to the fact that the ion-exchange membrane exposed to the anolyte in this system is cation-selective, *i.e*., CEM, and LC solution flows on opposite side of the CEM (for more details see Supplementary Fig. S1). Although water oxidation at the anode makes the anolyte acidic, the protons produced leak through the CEM to the LC compartment, mitigating a severe drop in pH. However, the water reduction reaction significantly changes the pH of catholyte that faces the other CEM and lies beyond the HC compartment. In this configuration, the penetration of the hydroxide ions produced, through the CEM into the HC compartment, is limited. The pH changes in the anolyte and catholyte are expected to be addressed in a further study.

## Conclusions

In summary, we have demonstrated a unique solar water-splitting system composed of a PEC cell and a RED stack. The RED stack, positioned between the electrodes of the PEC-RED hybrid system, serves not only as a separator but also as an additional tunable power source to compensate for the insufficient photovoltage produced by the photoelectrode. Water splitting is possible through the use of a standalone PEC cell^44–46^. However, no suitable photoelectrodes have yet been found for use in solar water splitting, and most research to date has focused on seeking new materials and/or structures. Our introduced concept provides flexibility in photoelectrode material selection, such as low-cost and reliable materials having narrow band gap. Moreover, the feasibility of fine-tuning the overpotential, without any external electricity, provides an effective means of selectively synthesizing a targeted solar fuel. This represents another step forward to cost-effective purification and preconcentration for practical use.

Considering the power consumed by the pump, the energy conversion efficiency of the PEC-RED system is about 0.55-1.8%. This value is comparable to the efficiency of a two-junction tandem PEC system (with two voltage sources) consisting of all earth-abundant material (for more details see Supplementary Table S3)^47^. To demonstrate the proof-of-concept that RED can be used as an auxiliary power source in a solar water splitting system, we conducted experiments under difficult conditions that required the significant external voltage. We expect the efficiency to be noticeably improved by optimizing the number of membrane-pairs, depending on the band gap of photoelectrode, the kinetic overpotentials required for the electrocatalyst, and the pH of electrolyte. The number of membrane-pairs influences the optimum conditions for the area ratio of the photoelectrode to the ion-exchange membrane, the flow rate, and the operating current. The integration of a PEC cell with a RED stack, to form a suitable hybrid system, offers new opportunities for tackling the challenges associated with solar-to-fuel conversion.

## Materials and Methods

### Fabrication of electrodes

The photocathode was fabricated on p-type Si (100) wafer ($1 \sim 10\,{\rm{ohm}}\cdot {\rm{cm}}$, Silicon Technology, Japan). To make a backside ohmic contact, 300 nm Al was deposited on the back surface of the Si wafer, followed by annealing at 400 °C for 30 s^48^. The NiMo electrocatalyst was electrodeposited from a sulfamate plating solution that contained 325 g L^−1^ Ni(SO_3_NH_2_)_2_, 5 g L^−1^ Na_2_MoO_4_, and 30 g L^−1^ H_3_BO_3_^31^. Electrodeposition was carried out using a potentiostat (CHI601C, CHInstrument) in three-electrode configuration with Pt mesh counter electrodes and Ag/AgCl (3 M NaCl, *E*° = + 0.209 V *vs*. NHE) reference electrode. For deposition, the Si electrode was held at −1.3 V vs. Ag/AgCl for 1 min under intense illumination. Pt NP was deposited onto Si electrode by electroless deposition. For the electroless deposition of Pt, Si electrode was immersed into a solution of 1 mM K_2_PtCl_6_ in 0.4 M HF for 2 min, followed by a rinse with $18.2\,{\rm{M}}{\rm{\Omega }}\cdot {\rm{cm}}$ deionized water (Millipore)^48^. The deposition process was repeated to increase the loading amount of Pt. The highest catalytic activity was observed after two iterations. Ni foam (>95% porosity) was purchased from MTI Korea and used after cleaning in a ultrasonic bath with acetone and isopropyl alcohol for 15 min each, followed by dry at 60 °C in an electric oven.

### Material characterizations

The electrodes were characterized by a field-emission scanning electron microscope (FE-SEM, SIGMA), an atomic force microscope (AFM, XE-150), X-Ray Photoelectron Spectroscopy (XPS, AXIS-HSi), and X-Ray Fluorescence Spectroscopy (XRF, XRF-1800).

### Photoelectrochemical measurements

Photoelectrochemical measurement was carried out using a potentiostat (CHI601C, CHInstrument) in two- or three-electrode configuration with Pt mesh counter electrodes and Ag/AgCl (3 M NaCl, *E*° =+0.209 V *vs*. NHE) reference electrode. All electrochemical experiments were performed in 0.1 M phosphate buffer (pH 7.0) with 0.5 M Na_2_SO_4_. A home-built solar simulator that had 150 W Xenon arc lamp with AM 1.5 G output was used as an illumination source. The intensity was calibrated to 100 mW cm^−2^ with a radiometer (PMA-2100, Solar light) and a pyranometer (PMA-2144). Characterizations of each electrode were conducted in a three-electrode configuration with Ag/AgCl reference electrode. Overall water splitting was tested in a two-electrode configuration. For gas quantification, the cathode and anode were immersed in 50 mL of 0.1 M phosphate buffer (pH 7.0) under a quartz vessel. After sealing tightly, Ar purging was conducted for 15 min for degassing. During continuous irradiance, the quartz cell with solar simulator (HAL-302, Ashai Spectra) under 100 mW cm^−2^ of light density, the voltage of 2.65 V was applied across the electrodes by potentiostat (CHI760E, CHInstrument). The evolved H_2_ and O_2_ are quantified by on-line gas chromatography (DS Science, iGC7200) with thermal conductivity detector (TCD) for every 5 min after a period of 50 min for stabilization.

### Preparation and characterization of RED stack

We utilized a RED stack configuration that is previously reported by B. E. Logan and M. C. Hatzell^49–51^. The RED stack is situated between the cathode and anode chamber. Gaskets between membranes (Selemion CMV and AMV) separate the membranes and create compartments filled with high- and low-concentration salt solution. Each compartment has a dimension of 4 cm × 2 cm × 1.3 mm. A unit structure of RED stack is composed of CEM/a compartment with HC/AEM/a compartment with LC/CEM (HC: 35 g L^−1^ of high-concentration salt solution, LC: 0.7 g L^−1^ of low-concentration salt solution). Overall RED stack was assembled by stacking the unit structures. HC flows from the cathode chamber to anode chamber through every HC compartment, and similarly LC flows but in the opposite direction to HC (for more details see Supplementary Fig. S1). Each solution was fed into RED stack at constant flow rate of 9 mL min^−1^ using a peristaltic pump. The characterization of RED performance was conducted using a potentiostat (CHI601C, CHInstrument) in two-electrode configuration with Ag/AgCl electrodes immersed in 0.5 M NaCl electrolyte. Electrochemical impedance analysis was carried out using a Gamry Reference 600 (Gamry Instruments) while applying a current density of 0.2 mA cm^−2^ as a DC current. Resistance of RED stack were determined at the frequency of 1 kHz. The power required for pumping was calculated through hydrodynamic loss across RED stack^43^. A pressure drop across the RED stack was measured by a manometer (HHP91, Omega).

### Data availability

The authors declare that all data supporting the findings of this study are available within the paper and its supplementary information file.

## Electronic supplementary material

Supplementary Information
