# Supplementary material for: Reverse Electrodialysis-Assisted Solar Water Splitting
Source: Sci Rep. 2017 Sep 25;7:12281. doi: 10.1038/s41598-017-12476-3 (PMC5612967; doi:10.1038/s41598-017-12476-3)
Supplement: Supplementary file 1 — Supplementary Information [file 41598_2017_12476_MOESM1_ESM.pdf]

Supplementary Information

Reverse Electrodialysis-Assisted Solar Water Splitting

Jihye Lee, Jeongse Yun, Seung-Ryong Kwon, Woo Je Chang, Ki Tae Nam,and Taek Dong Chung*

*To whom correspondence should be addressed. Email: tdchung@snu.ac.kr.

Table of Contents

Supplementary Figure S1. Schematic representation of reverse electrodialysis (RED) 2

Supplementary Figure S2. Characterization of NiMo/Si photocathode 4

Supplementary Figure S3. Optimization of NiMo electrodeposition time 5

Supplementary Figure S4. Characterization of Ni foam anode 6

Supplementary Figure S5. Effects of flow rate on open-circuit voltage (OCV) and resistance of RED stack with 16 membrane-pairs 7

Supplementary Figure S6. Photograph of the experimental set-up 8

Supplementary Figure S7. Current density-voltage curves as a function of flow rate 9

Supplementary Table S1. Hydrodynamic loss in RED stack 10

Supplementary Table S2. Energy conversion efficiency (η) 11

Supplementary Table S3. Previously reported solar water splitting system composed of a two-junction tandem configuration 12

Supplementary Note 12

Supplementary References 13

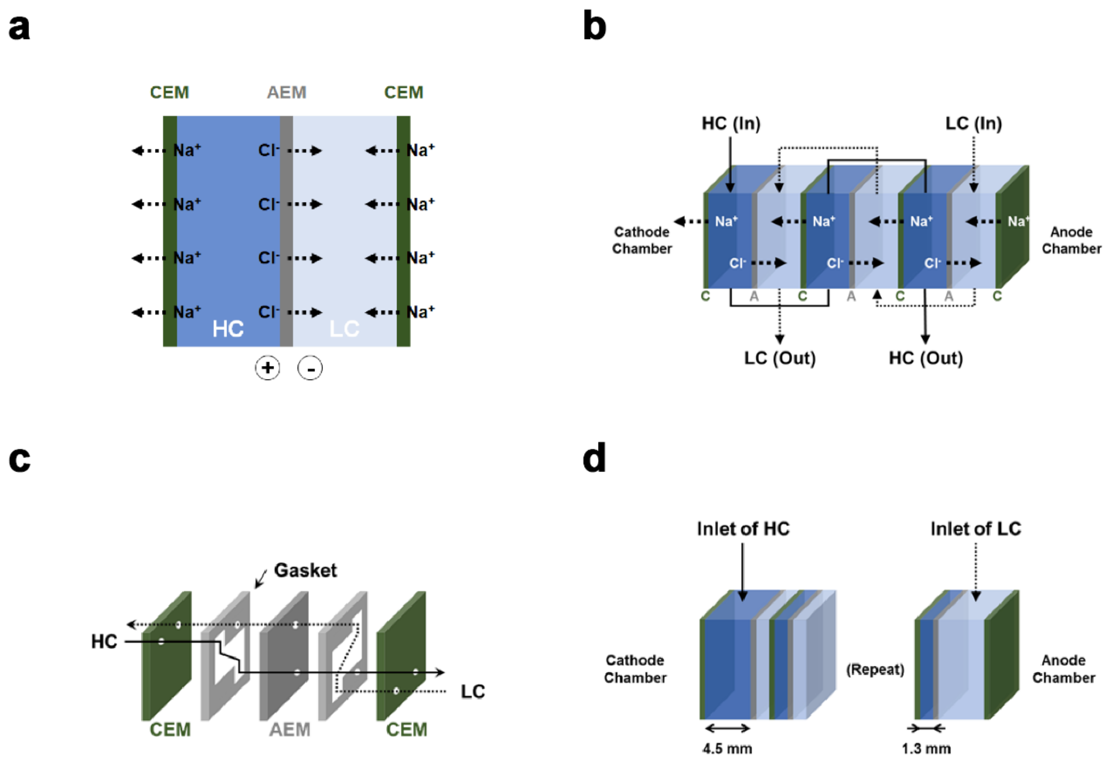

Supplementary Figure S1. Schematic representation of reverse electrodialysis (RED). (A) Junction potential across an ion-exchange membrane. CEM and AEM mean the cation-exchange membrane and the anion-exchange membrane, respectively. HC stands for a high-concentration solution and LC a low-concentration solution. (B) Structure of the RED stack. An ionic flux (*i.e.* Na^+ and Cl^-), which is generated inside the RED stack, is converted into an electricity or fuels at electrodes through an electrochemical reaction. In the PEC-RED hybrid system, the water-splitting reaction takes place at the electrodes and the hydrogen fuel is produced. (C) Flow path of the HC and LC solutions inside the RED stack. The gaskets, between the CEMs and the AEMs, separate the membranes and create a flow path of the HC and the LC solutions. When the HC solution flows in an HC compartment, the LC solution passes the HC compartment through a hole in a gasket, and vice versa. (D) The dimension of the HC and LC compartments inside RED stack.

When solutions with different ion concentrations flow on the opposite sides of an ion-exchange membrane, an electrochemical potential difference develops across the membrane by the chemical potential difference between the solutions.

The electrochemical potential for an ionic species i in phase α is defined as:

$$\overline{\mu}_{\mathrm{i}}^{\alpha} = \mu_{\mathrm{i}}^{0\alpha} + RT \ln a_{\mathrm{i}}^{\alpha} + z_{\mathrm{i}} F \phi^{\alpha} \qquad (1)$$

$\overline{\mu}_{\mathrm{i}}^{0\alpha}$ is the standard chemical potential, *R* is the gas constant, *T* is the absolute temperature, a_{i}^{α} and z_{i} are the activity and charge of the ionic species i, *F* is the Faraday constant, and ϕ is the electric potential [1].

The magnitude of the electric potential difference (or the junction potential) created across the ion-exchange membrane under the open-circuit condition is expressed as:

$$\left|\Delta\phi_{j}\right| = \frac{RT}{zF}\left[t_{\mathrm{counter}}\ln\left(\frac{a_{\mathrm{counter}}^{\mathrm{HC}}}{a_{\mathrm{counter}}^{\mathrm{LC}}}\right) - t_{\mathrm{co}}\ln\left(\frac{a_{\mathrm{co}}^{\mathrm{HC}}}{a_{\mathrm{co}}^{\mathrm{LC}}}\right)\right] \quad (2)$$

The transference number (t_{i}) is defined as the fraction of the current carried by the ion species i to the total electric current across the membrane. The “counter” and “co” mean counter- and co-ion, in the terms of the ion charge, relative to the fixed charge in the membrane [1].

Activity of the ionic species (a_{i}) is obtained by multiplying the molar concentration by the activity coefficient. The activity coefficient (γ_{i}) is calculated from the Debye-Hückel extended equation:

$$\log\gamma_{\mathrm{i}} = -\frac{A|z_{\mathrm{i}}|^{2}\sqrt{I}}{1+Ba^{0}\sqrt{I}} - \log(1+0.001m_{\mathrm{i}}M_{\mathrm{S}}) + C_{\mathrm{i}}I \quad (3)$$

A and B are the Debye-Hückel parameters, a^{0} is the ion size parameter (Å), I is the ionic strength in molality (m), M_{S} is the molecular mass of the solvent, and C is the ion-interaction parameter. For aqueous NaCl solution, when *T*=298.15 K, A is 0.5085 $\mathrm{mol^{-1/2}\,kg^{1/2}}$ and B is 0.3282 $\mathrm{Å^{-1}\,mol^{-1/2}\,K^{1/2}}$. a^{0} is 3.78 Å for both Na^{+} and Cl^{-}, M_{S} is 18.02 g $\mathrm{mol^{-1}}$, *C* is 0.105 $\mathrm{kg^{2}\,mol^{-2}}$ for Na^{+} ion and -0.009 $\mathrm{kg^{2}\,mol^{-2}}$ for Cl^{-}. This Debye-Hückel extended equation is valid up to about 1 mol $\mathrm{kg^{-1}}$ [2].

Assuming ideal ion-exchange membranes (t_{counter} is unity), the junction potential is about 0.19 V for a membrane-pair when the concentrations of HC and LC solutions are 35 g $\mathrm{L^{-1}}$ and 0.7 g $\mathrm{L^{-1}}$, respectively. Considering the chemical potential difference between the HC and LC solutions is 0.19 V, the chemical potential difference driven by salinity gradient is totally converted into the electric potential. In the actual ion-exchange membranes, the junction potential is predicted to 0.17 V based on Selemion cation- (CMV) and anion- (AMV) exchange membrane with transference number of > 0.96 for counter ions. These values are in good agreement with the experimentally measured values (for more details see main text Fig. 3**a**).

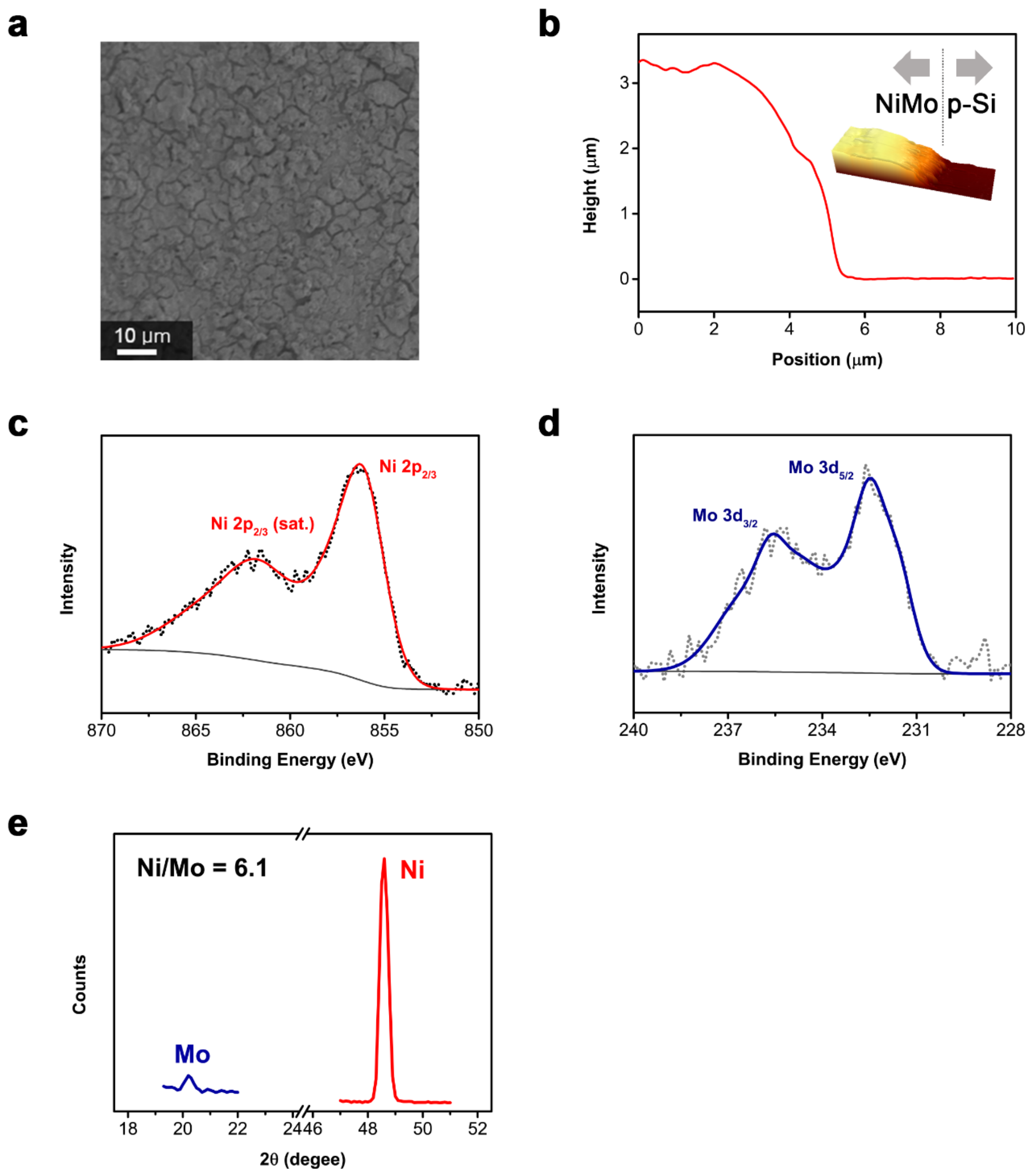

Supplementary Figure S2. Characterization of NiMo/Si photocathode. (A) Morphology, (B) AFM image, (C, D) XPS spectra, and (E) XRF spectra of a NiMo electrocatalyst film deposited on a p-Si surface.

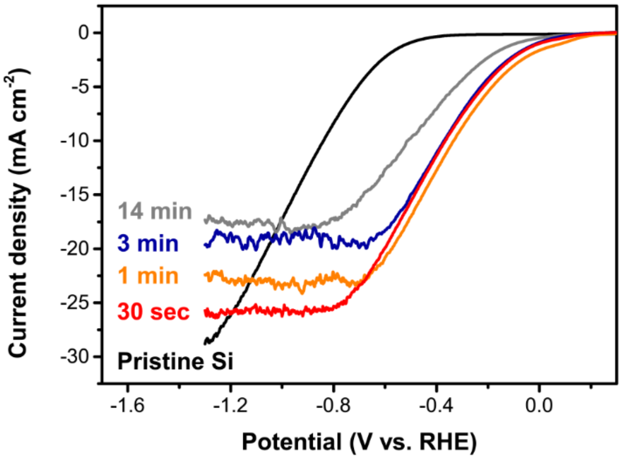

Supplementary Figure S3. Optimization of NiMo electrodeposition time. The current density–potential curves show that optimal time for NiMo electrodeposition was determined at 1 min.

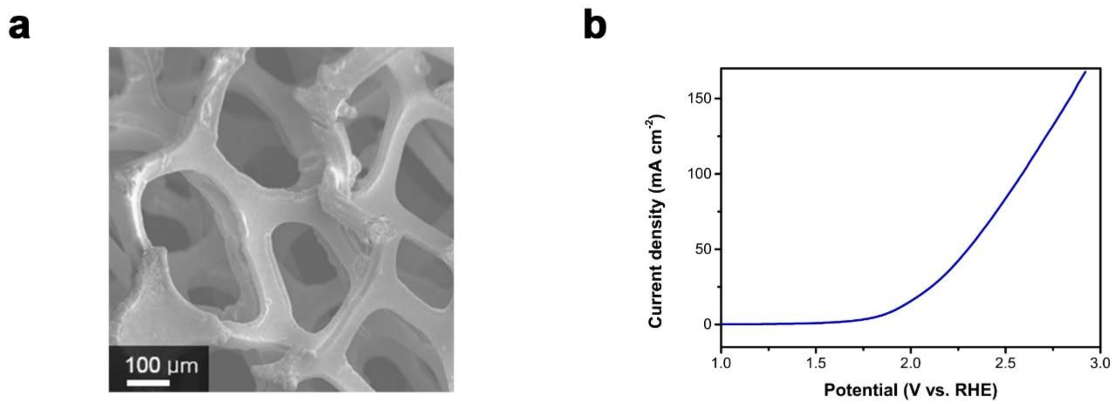

Supplementary Figure S4. Characterization of Ni foam anode. (A) Morphology and (B) oxygen evolving performance of Ni foam.

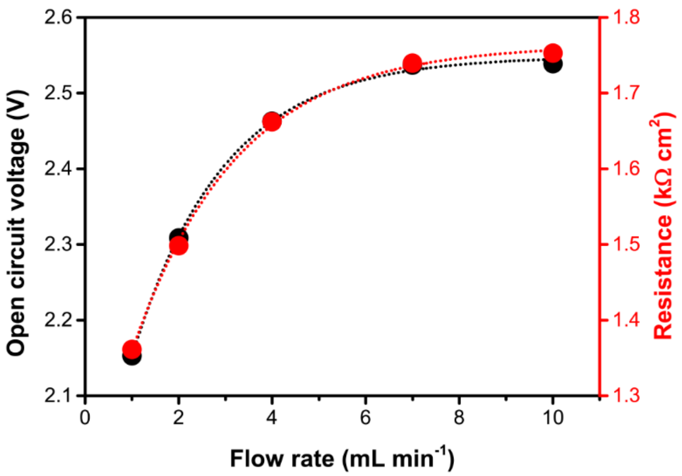

Supplementary Figure S5. Effects of flow rate on open-circuit voltage (OCV) and resistance of RED stack with 16 membrane-pairs. The flow rate influences the residence time of the HC and the LC solutions inside the RED stack.

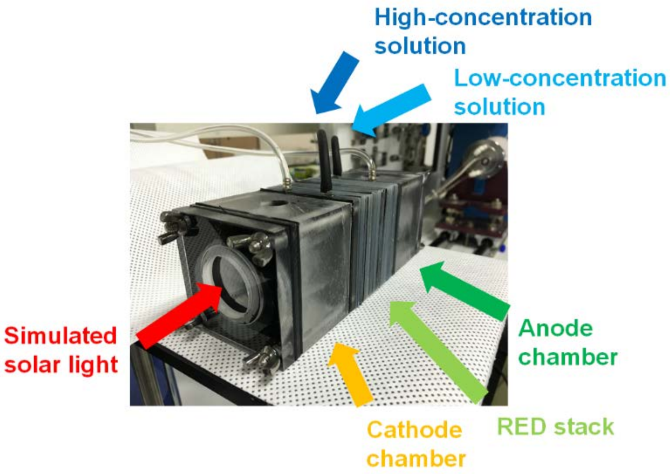

Supplementary Figure S6. Photograph of the experimental set-up. The overall water splitting cell was constructed using NiMo/Si and Ni foam as hydrogen- and oxygen-evolving electrodes, respectively. Simulated solar light was illuminated from the left side. The RED stack, situated between the two electrodes, acts as an additional power source, as well as a salt bridge that separates the catholyte from the anolyte. The HC and LC solutions are fed into the RED stack using a peristaltic pump.

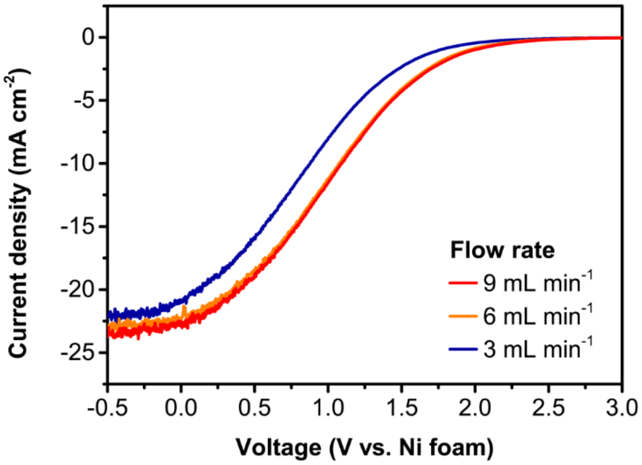

Supplementary Figure S7. Current density-voltage curves as a function of flow rate. There is a little difference in current density under zero bias condition when the flow rate decreases.

Supplementary Table S1. Hydrodynamic loss in RED stack.

Flow rate (mL min^{-1})	HC (Pa)	LC (Pa)	P_{hydro} (mW)	$P_{hydro}/P_{water\ splitting}$
9	1300	850	0.322	0.19
6	1100	650	0.175	0.10
3	1000	500	0.075	0.04

Notes: Hydrodynamic power loss (P_{hydro}) is defined as:

$$P_{\mathrm{hydro}} = \Phi_{\mathrm{HC}}\Delta P_{\mathrm{HC}} + \Phi_{\mathrm{LC}}\Delta P_{\mathrm{LC}} \quad (4)$$

Φ is the flow rate of HC and LC solutions ($\mathrm{L}\ s^{-1}$) and ΔP is the pressure drop (Pa) over the RED stack [3].

The H_2 chemical power produced ($P_{\mathrm{water\ splitting}}$) is expressed as:

$$P_{\mathrm{water\ splitting}} = [\ |j_{\mathrm{SC}}\ (\mathrm{mA\ cm^{-2}})|\ \times (1.23\ \mathrm{V})\ \times\ \varepsilon_{\mathrm{F}}\ ]_{\mathrm{AM\ 1.5\ G}} \quad (5)$$

j_{SC} is the short-circuit photocurrent density and ε_{F} is the faradaic efficiency for hydrogen- and oxygen-evolving reactions [4].

Supplementary Table S2. Energy conversion efficiency (*η*).

Flow rate (mL min^{-1})	η (%)
9	0.55
6	0.90
3	1.84

Notes: Energy conversion efficiency (η) is calculated from the ratio of the net produced power to the total input power as:

$$\eta = \left(\frac{P_{\text{water splitting}} - P_{\text{hydro}}}{P_{\text{input}}}\right) \times 100 \qquad (6)$$

The total system output power (P_{input}) is the sum of the solar power (100 mW cm^{-2}) and the salinity driven power.[3,5]

The salinity-driven power is calculated from the change in the Gibbs free energy when the HC and LC are completely mixing as[3]

$$P_{\text{Salinity}} = 2\Phi RT\left(c_{\text{LC}}\ \ln\frac{a_{\text{LC}}}{a_{\text{mixed}}} + c_{\text{HC}}\ \ln\frac{a_{\text{HC}}}{a_{\text{mixed}}}\right) \qquad (7)$$

Supplementary Table S3. Previously reported solar water splitting system composed of a two-junction tandem configuration.

Electrode (cathode // anode)	Electrolyte	Membrane	STH efficiency	Stability	Reference
p-Si with Pt // n-$BiVO_4$ with Co-Pi	0.1 M PB* (pH 5.5)	w/o	0.57%	3.5 h	6
a-pin-Si with TiO_2/Pt // n-Fe_2O_3 with $NiFeO_x$	0.5 M PB (pH 11.8)	w/o	0.91%	10 h	7
Ti with NiMo // GaAs/InGaP/TiO_2 with Ni	1.0 M KOH	AEM†	8.6%	>40 h	8
Pt // p-Si/n-Fe_2O_3 with Au	1 M Na_3PO_4	w/o	6.0%	40 min	9
Pt // np^+-Si/n-WO_3	1 M H_2SO_4	Nafion	0.0068%	10 min	10
Si solar cell with Ni // Mo:$BiVO_4$ with FeOOH	0.5 M PB (pH 7)	w/o	2.52%	10 h	11
pn^+-Si with Pt // n-TiO_2 with IrO_x	0.5 M H_2SO_4	w/o	0.12%	4.5 h	12
Pt // DSSC‡/n-WO_3	1 M $HClO_4$ (pH 0)	w/o	3.1%	-	13
Pt // DSSC/n-Fe_2O_3 with Al_2O_3/Co	1 M NaOH (pH 13.6)	w/o	1.17%	8 h	13
p/n-GaAs/p-$GaInP_2$ with Pt // Pt	3 M H_2SO_4	w/o	12.4%	20 h	14
**p-Si with NiMo // Ni**	**0.1 M PBS§ (pH 7.0)**	**IEMs‖**	**0.55-1.84%**	**25 h**	**This study**

* Phosphate buffer solution.

† Anion-exchange membrane.

‡ Dye-sensitized solar cell.

§ 0.1 M phosphate buffered saline with 0.5 M Na_2SO_4.

‖ Ion-exchange membranes.

Supplementary Note

Series connection between NiMo/Si photoelectrode and RED stack

In a series connection (or series circuit), electrons (or current) flow in a single path in a circuit, and the voltage across the circuit is the sum of the circuit components. When the NiMo/p-Si photocathode absorbs solar light, photo-excited electrons and holes are generated in the electrode. The electrons are transported to the semiconductor-electrolyte interface and reduce the water to H_2. In the electrolyte, ions are responsible for charge transfer instead of electrons. On the catholyte side, the anions (such as SO_4^{2-}, $H_2PO_4^-$ and HPO_4^{2-}) move toward the RED stack and the cations (such as Na^+) towards the photoelectrode. Similar to the charge transfer in the electrolyte, Na^+ and Cl^- ions carry charges inside the RED stack. On the anolyte side, the anions are migrated towards the Ni foam anode, where charges (electrons) are transferred to the anode by the water oxidation reaction. The electrons move along the conductive wire from the anode to the photocathode and are recombined with the holes at the photoelectrode to form a closed circuit.

Energy diagram of the PEC-RED system

The NiMo/Si photocathode and the Ni foam anode have a leveled Fermi level because they are connected by a conductive wire. Under illumination, the concentration of minority carriers (electrons) increases and thus the quasi-Fermi level of the electrons is separated from that of the holes at the photocathode. When the quasi-Fermi level of the electrons is more negative than the thermodynamic water reduction potential (on the electrochemical scale), the hydrogen evolution reaction occurs. The RED stack builds an electric field in the electrolyte, which changes the relative position of the water reduction/oxidation potential. As a results, the thermodynamic water oxidation potential is more negative than the Fermi level of the electrons in the anode and thus oxygen evolution reaction occurs.

Economic issues of the PEC-RED system

In the PEC-RED system, it is relatively easy to obtain high voltage by increasing the number of membranes. But the power density is low because of the high resistance of the RED stack. Therefore, the most of the voltage drop occurs across the RED stack when the current increases. Consequently, this system is not suitable for situations where a high current flows. Moreover, it is estimated that membranes occupy up to 80% of total capitals in salinity gradient-based energy generation systems including RED.[15] Therefore, the feasibility of PEC-RED system depends mainly on the membrane price. Since the currently available commercial membranes are developed for use in electrodialysis, they have high level of robustness and are rather thick. However, the commercial membranes for electrodialysis are overqualified for application in RED because the

conditions where RED operates is relatively mild. In order to implement RED stack to PEC cell, a thinner ion-selective membranes having low resistance, high permselectivity and excellent mechanical stability should be developed. An economical membrane is about 4 € m^{-2} but low-resistance membrane is still expensive (>80 € m^{-2}).[16]

Supplementary References

1. Bard, A. J. & Faulkner, L. R. *Electrochemical Methods: Fundamentals and Applications*. (John Wiley & Sons, Inc., 2000).
2. Zhuo, K., Dong, W., Wang, W. & Wang, J. Activity coefficients of individual ions in aqueous solutions of sodium halides at 298.15K. *Fluid Phase Equilib.* **274,** 80–84 (2008).
3. Veerman, J., Saakes, M., Metz, S. J. & Harmsen, G. J. Electrical Power from Sea and River Water by Reverse Electrodialysis: A First Step from the Laboratory to a Real Power Plant. *Environ. Sci. Technol.* **44,** 9207–9212 (2010).
4. Chen, Z. *et al.* Accelerating materials development for photoelectrochemical hydrogen production: Standards for methods, definitions, and reporting protocols. *J. Mater. Res.* **25,** 3–16 (2011).
5. Coridan, R. H. *et al.* Methods for comparing the performance of energy-conversion systems for use in solar fuels and solar electricity generation. *Energy Environ. Sci.* **8,** 2886–2901 (2015).
6. Xu, P. *et al.* Photoelectrochemical cell for unassisted overall solar water splitting using a BiVO 4 photoanode and Si nanoarray photocathode. *RSC Adv.* **6,** 9905–9910 (2016).
7. Jang, J.-W. *et al.* Enabling unassisted solar water splitting by iron oxide and silicon. *Nat. Commun.* **6,** 7447 (2015).
8. Verlage, E. *et al.* A monolithically integrated, intrinsically safe, 10% efficient, solar-driven water-splitting system based on active, stable earth-abundant electrocatalysts in conjunction with tandem III–V light absorbers protected by amorphous TiO 2 films. *Energy Environ. Sci.* **8,** 3166–3172 (2015).
9. Wang, X. *et al.* Silicon/hematite core/shell nanowire array decorated with gold nanoparticles for unbiased solar water oxidation. *Nano Lett.* **14,** 18–23 (2014).
10. Shaner, M. R. *et al.* Photoelectrochemistry of core–shell tandem junction n–p + -Si/n-WO 3 microwire array photoelectrodes. *Energy Environ. Sci.* **7,** 779–790 (2014).
11. Ding, C. *et al.* Solar-to-hydrogen efficiency exceeding 2.5% achieved for overall water splitting with an all earth-abundant dual-photoelectrode. *Phys. Chem. Chem. Phys.* **16,** 15608 (2014).
12. Liu, C., Tang, J., Chen, H. M., Liu, B. & Yang, P. A Fully Integrated Nanosystem of Semiconductor Nanowires for Direct Solar Water Splitting. *Nano Lett.* **13,** 2989–2992 (2013).
13. Brillet, J. *et al.* Highly efficient water splitting by a dual-absorber tandem cell. *Nat. Photonics* **6,** 824–828 (2012).
14. Khaselev, O. & Turner, J. a. A Monolithic Photovoltaic-Photoelectrochemical Device for Hydrogen Production via Water Splitting. *Science (80-.).* **280,** 425–427 (1998).
15. Ruud Kempener & Neumann, F. Salinity Gradient Energy Technology Brief. in *IRENA Ocean Energy Technology Brief 2* 1–28 (IRENA, 2014).
16. Guler, E., Zhang, Y., Saakes, M. & Nijmeijer, K. Tailor-Made Anion-Exchange Membranes for Salinity Gradient Power Generation Using Reverse Electrodialysis. *ChemSusChem* **5,** 2262–2270 (2012).
